# On the Plurality of (Methodological) Worlds: Estimating the Analytic Flexibility of fMRI Experiments

**DOI:** 10.3389/fnins.2012.00149

**Published:** 2012-10-11

**Authors:** Joshua Carp

**Affiliations:** ^1^Department of Psychology, University of MichiganAnn Arbor, MI, USA

**Keywords:** fMRI, data analysis, analysis flexibility, selective reporting, false positive results

## Abstract

How likely are published findings in the functional neuroimaging literature to be false? According to a recent mathematical model, the potential for false positives increases with the flexibility of analysis methods. Functional MRI (fMRI) experiments can be analyzed using a large number of commonly used tools, with little consensus on how, when, or whether to apply each one. This situation may lead to substantial variability in analysis outcomes. Thus, the present study sought to estimate the flexibility of neuroimaging analysis by submitting a single event-related fMRI experiment to a large number of unique analysis procedures. Ten analysis steps for which multiple strategies appear in the literature were identified, and two to four strategies were enumerated for each step. Considering all possible combinations of these strategies yielded 6,912 unique analysis pipelines. Activation maps from each pipeline were corrected for multiple comparisons using five thresholding approaches, yielding 34,560 significance maps. While some outcomes were relatively consistent across pipelines, others showed substantial methods-related variability in activation strength, location, and extent. Some analysis decisions contributed to this variability more than others, and different decisions were associated with distinct patterns of variability across the brain. Qualitative outcomes also varied with analysis parameters: many contrasts yielded significant activation under some pipelines but not others. Altogether, these results reveal considerable flexibility in the analysis of fMRI experiments. This observation, when combined with mathematical simulations linking analytic flexibility with elevated false positive rates, suggests that false positive results may be more prevalent than expected in the literature. This risk of inflated false positive rates may be mitigated by constraining the flexibility of analytic choices or by abstaining from selective analysis reporting.

## Introduction

How common are false positive results in the functional neuroimaging literature? Among functional MRI (fMRI) studies that apply statistical correction for multiple comparisons, most use a nominal false positive rate of 5%. However, Wager et al. (2009) estimate that between 10 and 40% of fMRI activation results are false positives. Furthermore, recent empirical (Ioannidis, 2005a) and mathematical modeling studies (Ioannidis, 2005b) argue that the true incidence of false positives may far exceed the nominal rate in the broader scientific literature. Indeed, under certain conditions, research findings are more likely to be false than true (Ioannidis, 2005b).

As described in a mathematical modeling study by Ioannidis (2005b), analytic flexibility is a key risk factor for inflated rates of false positive results when combined with selective reporting of favorable analysis methods. Analytic flexibility is defined here as the range of analysis outcomes across different acceptable analysis methods. Thus, if many analysis pipelines are considered valid, and if different methods yield different results, then analysis flexibility is high. When analytic flexibility is high, investigators may elect to report methods that yield favorable outcomes and omit methods that yield null results. This practice is known as selective analysis reporting. For example, a researcher may notice that an experiment yields positive results when analyzed using head motion regression, but not when analyzed without using head motion regression. The researcher may then choose to describe the former analysis but not the latter when reporting the results of the experiment. Indeed, investigators in some research fields appear to pursue this strategy. Reviews of randomized clinical trials show that many studies change outcome measures and other methodological parameters between study design and publication. Critically, these changes tend to make results appear more significant than they would have been under the original analysis plan (Chan et al., 2004a,b; Dwan et al., 2008; Mathieu et al., 2009).

A recent survey of fMRI methods shows that methodological decisions are highly variable from study to study (Carp, 2012). Across 241 published fMRI studies, authors reported using 32 unique software packages (e.g., SPM 2, FSL 3.3) and 207 unique combinations of design and analysis steps (e.g., spatial normalization, head motion regression). Parameter settings also showed considerable variability within each analysis step. For example, spatial smoothing kernels ranged from 3 to 12 mm full width at half maximum, and high-pass filter cutoffs ranged from 0.33 to 750 s. Because many studies did not describe critical analysis decisions, this survey likely understated the true diversity of experimental methods in the fMRI literature. In other words, fMRI researchers may choose from a wide array of acceptable methodological strategies.

Critically, methodological studies suggest that this variability in analytic strategies may translate into variability in research outcomes. Countless studies show that individual methodological decisions can have important effects on estimates of fMRI activation. For example, temporal filtering (Skudlarski et al., 1999), autocorrelation correction (Purdon and Weisskoff, 1998; Woolrich et al., 2001), global signal regression (Murphy et al., 2009; Weissenbacher et al., 2009), and head motion regression (Friston et al., 1996; Lund et al., 2005) can profoundly influence analysis outcomes. Activation estimates also vary with the order of analysis steps (Weissenbacher et al., 2009; Carp, 2011) and across analysis software packages (Smith et al., 2005; Poline et al., 2006). Further, combinations of analysis decisions may have interactive effects on research outcomes (Churchill et al., 2012a,b).

However, while many studies have examined the effects of individual analysis procedures or combinations of procedures on research outcomes, most of these studies have focused on optimizing the selection of analytic pipelines rather than quantifying variability across pipelines. For example, Skudlarski et al. (1999) investigated variations between analysis pipelines in receiver operating characteristic (ROC) measures; Della-Maggiore et al. (2002) assessed the effects of differing pipelines on statistical power; and Strother and colleagues (Strother et al., 2004; Churchill et al., 2012a,b) evaluated pipelines using reproducibility and prediction metrics. However, while these studies offer valuable insights into which procedures should be applied and which parameters should be used, they did not explicitly assess the variability of research outcomes across analysis pipelines. In contrast, Hopfinger et al. (2000) did measure variability in activation amplitude across 36 distinct pipelines. But this study examined just four analysis steps, rather than the complete pre-processing and modeling pipelines used in most current fMRI studies, and focused on regional rather than whole-brain activation results. Altogether, while a wealth of previous studies have investigated the question of pipeline optimization, relatively few have considered the question of pipeline variability.

Thus, expanding on previous studies of analytic flexibility, the present study estimated the variability of fMRI methods across 10 pre-processing and model estimation steps. Between two and four options were considered for each step (see Tables 1 and 2). Enumerating all combinations of each of the steps yielded a total of 6,912 unique analysis pipelines. Activation estimates from each pipeline were then statistically thresholded and corrected for multiple comparisons using five commonly used techniques, yielding 34,560 unique thresholded activation maps. By examining a range of analysis pipelines orders of magnitude greater than those considered in previous studies, the present investigation yields the most comprehensive picture of methodological flexibility in the fMRI literature available to date.

**Table 1:** **Pre-processing parameters**.

**Despiking**			
Despiking using AFNI		No despiking	
**Slice-timing correction**			
Slice-timing correction		No slice-timing correction	
**Spatial normalization**			
Normalization of functional images to the SPM EPI template	Normalization of anatomical images to the SPM T1 template		Normalization with segmentation using unified normalization
**Spatial smoothing**			
Smoothing with kernel of 4 mm FWHM	Smoothing with kernel of 8 mm FWHM		Smoothing with kernel of 12 mm FWHM

**Table 2:** **Model estimation parameters**.

**Normalization-modeling order**			
Normalize before modeling		Model before normalization	
**High-pass filtering**			
High-pass filtering using a cutoff of 128 s		No high-pass filtering	
**Temporal autocorrelation correction**			
AR(1) modeling		No correction for temporal autocorrelation	
**Run concatenation**			
Runs concatenated before model estimation		No run concatenation	
**Model basis set**			
Hemodynamic response function	Finite impulse response^1^, time points 3–4 versus baseline		Finite impulse response^1^, time by condition interaction
**Head motion regression**			
Six regressors^2^	Twelve regressors^3^	Twenty-four regressors^4^	No motion regression

*^1^Eight basis functions*.

*^2^Raw motion parameters*.

*^3^Raw and time-shifted motion parameters*.

*^4^Raw, time-shifted, squared, and time-shifted squared motion parameters*.

## Materials and Methods

### Data acquisition

The present study re-analyzed a previously published fMRI study of response inhibition (Aron et al., 2007). Data were drawn from the Open fMRI database^1^ (Accession Number: ds000008; Task: 001). Fifteen subjects completed three runs of a standard event-related stop-signal task and three runs of a conditional stop-signal task. Only data from the standard stop-signal task were considered here. The task included three trial types. On go trials, subjects were instructed to make a motor response; on successful stop trials, subjects were instructed to withhold a response and were able to do so; and on failed stop trials, subjects were instructed to withhold a response but failed to do so. Functional data were acquired using a 3 T Siemens Allegra MRI scanner (TR: 2 s; TE: 30 ms; flip angle: 90°; voxel dimensions: 3.125 mm × 3.125 mm × 4.0 mm). Each of the three functional scanning runs included 176 images. High-resolution T1 MPRAGE images were also acquired for use in spatial normalization (TR: 2.3 s; TE: 2.1 ms; voxel dimensions: 1.0 mm × 1.33 mm × 1.33 mm). Complete imaging and behavioral data were only available for 13 of the subjects; the remaining two subjects were excluded from analysis. Further details on sample characteristics, task specifications, and imaging acquisition are given in the original report of these data (Aron et al., 2007).

### Pipeline generation

To generate a large collection of analysis pipelines, five pre-processing decisions and five modeling decisions for which multiple strategies appear in the research literature were selected. Pre-processing decisions, detailed in Table 1, included despiking (despiking or no despiking), slice-timing correction (slice-timing correction or no correction), spatial normalization (normalization to a functional template, to an anatomical template, or using segmentation of anatomical images), and spatial smoothing (FWHM 4, 8, or 12 mm). Modeling decisions, detailed in Table 2, included the order of normalization and model estimation (images were normalized before or after model estimation), high-pass filtering (128 s cutoff or no filtering), autocorrelation correction [AR(1) correction or no correction], run concatenation (run concatenation or no run concatenation), basis set [canonical hemodynamic response function, finite impulse response (FIR) with the contrast of time points 3 and 4 versus fixation, and FIR with the interaction of time point by condition], and head motion regression (6, 12, or 24 motion parameters, or no motion regression). Taking all combinations of these options yielded 6,912 unique analysis pipelines.

Despiking was implemented using the 3dDespike tool in AFNI version 2011_05_26_1456. All other steps were implemented using SPM 8 release 4010 (Wellcome Trust Centre for Neuroimaging, UCL, UK) running under Matlab 2011b (The Mathworks, Inc., Natick, MA, USA).

Data from each subject were submitted to each analysis pipeline. Each single-subject model included separate regressors for go trials, successful stop trials, and failed stop trials. Single-subject models were combined using random-effects analysis. Test statistics (i.e., *t* and *F* values) were converted to *Z*-values after contrast estimation using a transformation adapted from the ttoz and ftoz utilities in FSL version 4.1.8. All further analysis was based on random-effects models of the contrast of successful stop trials versus go trials.

To assess the variability in activation strength across models, the range of *Z*-values (referred to hereafter as the analytic range) was computed for each voxel and for each contrast. In addition, the range of activation values associated with each analysis step (despiking, slice-timing correction, etc.) was estimated by computing the mean absolute difference of *Z*-values over all pairs of parameter options and over all settings of other analysis parameters. For example, to estimate the analytic range attributable to changes in spatial smoothing kernel, the absolute value of the differences between (a) 4 and 8 mm FWHM, (b) 4 and 12 mm FWHM, and (c) 8 and 12 mm FWHM were averaged over all combinations of all other analysis parameters for each voxel and each contrast. Because the analytic range metric used here is based on variability in *Z*-values, this metric is sensitive to differences in both parameter estimates and error variance across pipelines.

Many neuroimaging studies report the locations of peak activation for contrasts of interest. Indeed, spatial precision is often advertised as one of the chief virtues of MRI as compared with other imaging techniques. Thus, the variability of peak activation coordinates across analysis pipelines was assessed as well. For each analysis pipeline and each contrast, the coordinates of the peak activation from each hemisphere were extracted. The distribution of peak coordinates was then plotted to assess the spatial dispersion of peak activation locations. To assess variability in localization within circumscribed regions of interest (ROIs), coordinates of peak activation were also extracted for each analysis pipeline within each of two ROIs: a right inferior frontal gyrus region (comprising the pars triangularis and pars opercularis regions of the right inferior frontal gyrus) and a right temporal cortex region (comprising the right superior and middle temporal gyri). All ROIs were defined using the Automatic Anatomical Labeling (AAL) atlas (Tzourio-Mazoyer et al., 2002).

The 6,912 random-effects statistical maps were also thresholded and corrected for multiple comparisons according to five strategies (Table 3), yielding 34,560 thresholded maps for each contrast. Activation maps were thresholded using three versions of a Monte Carlo simulation procedure, as implemented in the Resting-State fMRI Data Analysis Toolkit (REST; Song et al., 2011)^2^. These three thresholding approaches used uncorrected single-voxel thresholds of *p* < 0.01, *p* < 0.001, or *p* < 0.0001. Cluster size thresholds were then selected to set the cluster-wise false positive rate at 5% for each approach. Statistical maps were also thresholded using the false discovery rate (FDR; Genovese et al., 2002) and Gaussian random field theory (RFT; Nichols and Hayasaka, 2003) correction procedures, as implemented in SPM 8. Both the FDR and RFT procedures used a corrected single-voxel threshold of *p* < 0.05; neither of these methods employed cluster size thresholds.

**Table 3:** **Statistical thresholding parameters**.

	Uncorrected single-voxel threshold	Corrected single-voxel threshold	Cluster size threshold
Monte Carlo @ *p* < 0.01	*p* < 0.01	n/a	Determined by simulation
Monte Carlo @ *p* < 0.001	*p* < 0.001	n/a	Determined by simulation
Monte Carlo @ *p* < 0.0001	*p* < 0.0001	n/a	Determined by simulation
False discovery rate	n/a	*p* < 0.05	n/a
Gaussian random field theory	n/a	*p* < 0.05	n/a

It is important to note that these thresholding methods take different approaches to the problem of multiple comparisons. The Monte Carlo and RFT corrections used here attempt to control the family wise error at 5%. Using these corrections, 5% of activation maps should contain at least one false positive activation. In contrast, the FDR correction attempts to control the proportion of false positive voxels, such that 5% of significantly activated voxels should be false positives in a given activation map. Further, while the RFT and FDR corrections control the false positive rate at the level of individual voxels, the Monte Carlo correction controls the false positive rate at the level of clusters. Because these thresholding strategies approach the problem of multiple comparisons in different ways, it was expected that different strategies would yield different results. However, all three strategies appear to be used interchangeably in published studies, with many reports describing their chosen approach simply as “correcting for multiple comparisons.”

All code for generating analysis pipelines, calculating analytic variability, statistical thresholding, and plotting figures is freely available online^3^.

## Results

### Analytic variability of activation strength

**Figure 1:**
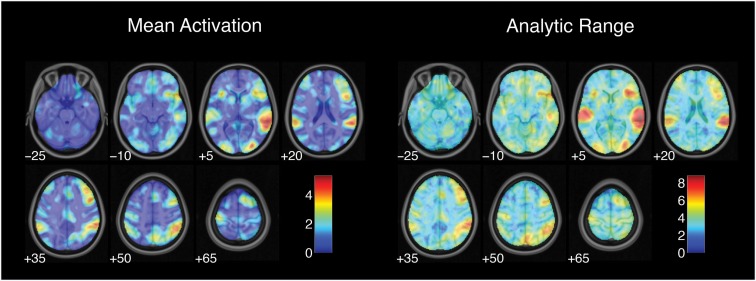
**Variation in activation strength across analysis pipelines**. Mean activation denotes the average *Z*-value for each voxel across all analysis pipelines; analysis range denotes the range of *Z*-values across all pipelines. Images are presented in neurological orientation, with the left hemisphere displayed on the left. Note that color scales differ across panels.

Estimates of activation strength showed substantial variability across analysis pipelines. Analytic range values (i.e., the range of *Z*-values across pipelines) for the contrast of successful stop trials versus go trials are displayed in Figure 1. Range values varied from 1.14 in the right superior frontal gyrus to 8.83 in the right superior temporal gyrus, with a median analytic range value of 3.44. Analytic range also varied with mean activation across analysis pipelines. Mean activation and analytic range for the successful stop versus go contrast were highly correlated across voxels [*r*(44,614) = 0.87, *p* < 0.001], such that voxels with the strongest activation also showed the greatest variability across analysis pipelines.

**Figure 2:**
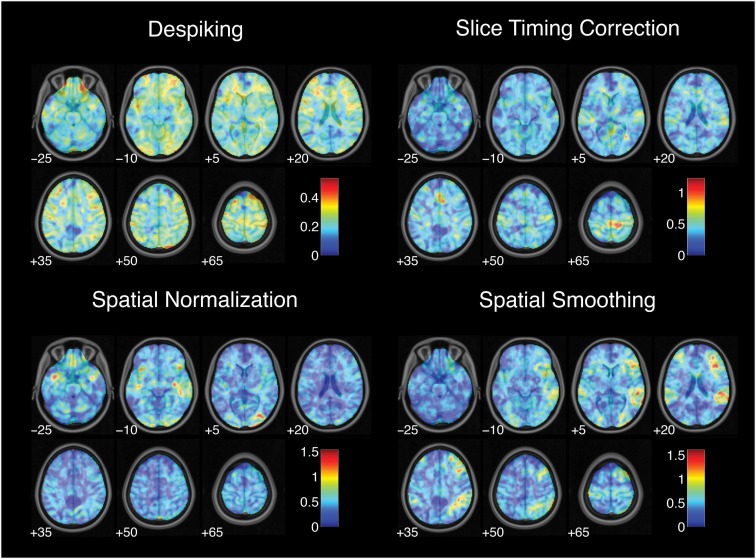
**Variation in activation strength attributable to pre-processing choices**. Images are presented in neurological orientation, with the left hemisphere displayed on the left. Note that color scales differ across panels.

**Figure 3:**
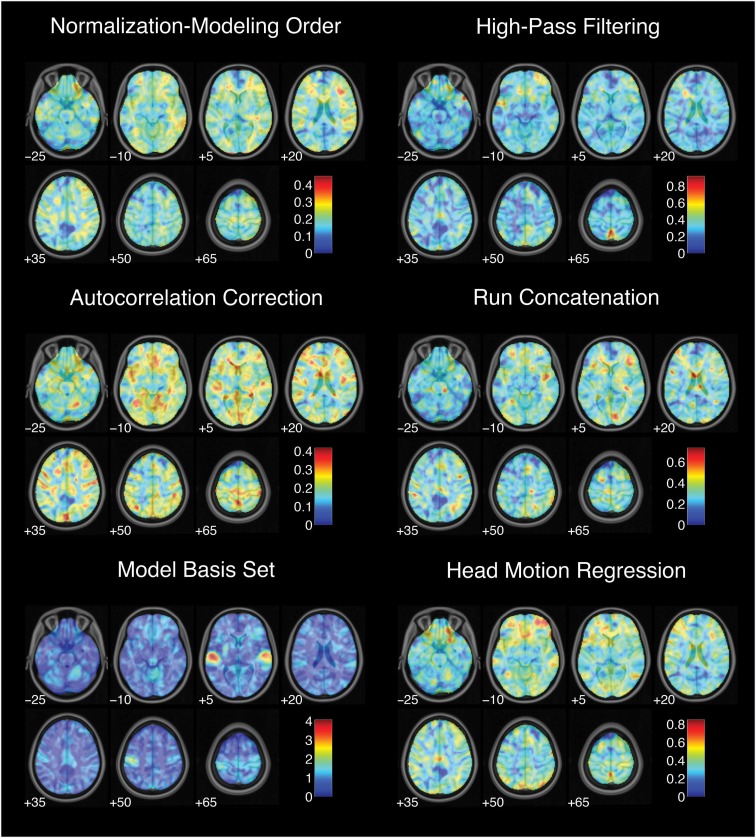
**Variation in activation strength attributable to model estimation choices**. Images are presented in neurological orientation, with the left hemisphere displayed on the left. Note that color scales differ across panels.

While each analysis step contributed to variability in activation strength across pipelines, different steps were associated with distinct patterns of variability across brain regions. For the contrast of successful stop trials versus go trials, the analytic range values for choices of smoothing kernel (Figure 2) and model basis set (Figure 3) were greatest in regions of maximal mean activation, including superior temporal gyrus and precuneus. In contrast, the effects of despiking (Figure 2) and head motion regression (Figure 3) were generally greatest toward the edges of the brain, particularly in ventral frontal regions. Other steps, such as slice-timing correction and spatial normalization, exerted idiosyncratic patterns of focal effects in a variety of regions across the brain (Figure 2), whereas autocorrelation correction was associated with diffuse patterns of change across the brain and ventricles (Figure 3).

Finally, range maps were moderately correlated across analysis steps. The mean absolute correlation across voxels between range maps for all pairs of analysis steps was *r* = 0.49, with an average explained variance of *R*^2^ = 0.26. In other words, while different analysis steps exerted spatially correlated effects on analysis outcomes across the brain, correlations among step-wise variability maps explained a minority of the variance associated with other analysis steps.

Thus, estimates of activation strength showed considerable variability across analytic pipelines; voxels that showed highly significant activations under some pipelines yielded null results under others. Pipeline-related variability was strongly correlated with average activation, such that activation estimates were most variable in regions showing the greatest overall activation. Finally, different analysis steps showed correlated but distinct patterns of influence across the brain.

### Analytic variability of activation location

**Figure 4:**
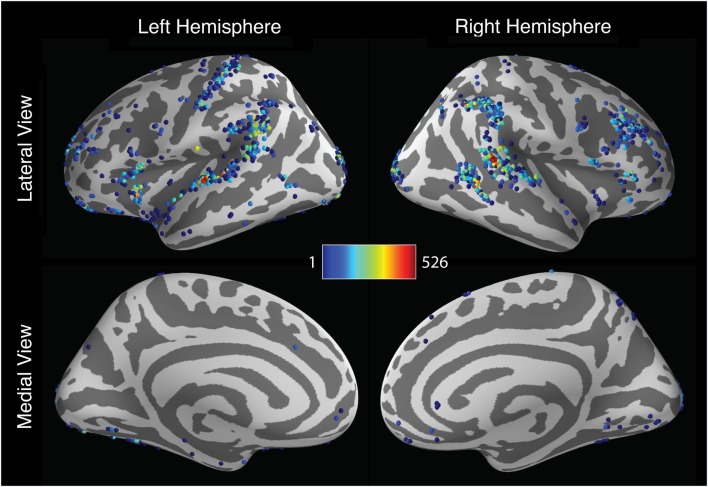
**Spatial distribution of peak activation locations across analysis pipelines across the cerebral hemispheres**. Shaded spheres indicate the locations of activation peaks. Sphere colors denote the base-10 logarithm of the number of pipelines yielding maximal activation for that location; colors range from blue, indicating a single pipeline, to red, indicating 526 pipelines.

Activation localization also varied widely across analysis pipelines. To describe the spatial dispersion of peak activation locations, the coordinates of the most significant activation were extracted for each hemisphere and for each pipeline. As seen in Figure 4, the results showed a considerable degree of consistency across pipelines: many pipelines yielded maximal activation in the superior temporal gyrus, the supramarginal gyrus, and the right inferior frontal gyrus. Within these regions, however, peak locations were widely dispersed, with activations extending along the length of the sylvian fissure. And many pipelines yielded peak locations outside these regions. In the left hemisphere, 672 unique peak locations were observed, with standard deviations of 12.8, 38.5, and 21.8 mm along the *x*-, *y*-, and *z*-axes, respectively. Activation peaks extended along the anterior-posterior axis from the middle frontal gyrus (*y* = 63.0) to the middle occipital gyrus (*y* = −108.875); along the lateral-medial axis from the middle temporal gyrus (*x* = −71.75) to the middle occipital gyrus (*x* = −18.625); and along the inferior-superior axis from the posterior cerebellum (*z* = −50) to the postcentral gyrus (*z* = 80.0). In the right hemisphere, 534 unique peaks were observed, with standard deviations of 12.6, 30.4, and 16.4 mm. Peaks ranged along the anterior-posterior axis from the superior frontal gyrus (*y* = 56.75) to the middle occipital gyrus (*y* = −108.875); along the medial-lateral axis from the posterior cerebellum (*x* = −15.5) to the superior temporal gyrus (*x* = 72.0); and along the inferior-superior axis from the posterior cerebellum (*z* = −50.0) to the postcentral gyrus (*z* = 75.0). In all, peaks were identified in 69 of the 128 regions defined by the AAL atlas.

**Figure 5:**
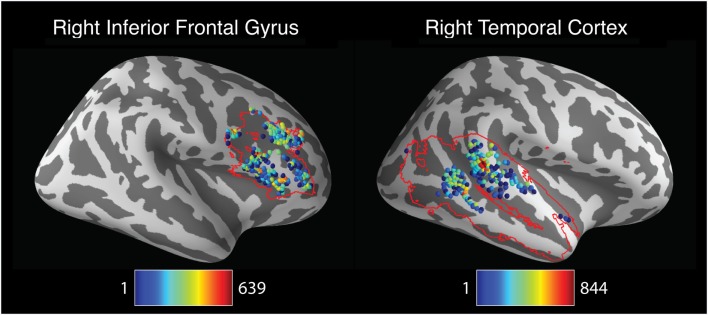
**Spatial distribution of peak activation locations across analysis within anatomically defined regions of interest (ROIs)**. Red contour lines indicate the boundaries of the ROIs. All images represent lateral views of the right hemisphere. Shaded spheres indicate the locations of activation peaks. Sphere colors denote the base-10 logarithm of the number of pipelines yielding maximal activation for that location. For the right inferior frontal gyrus ROI (left panel), colors range from blue, indicating a single pipeline, to red, indicating 639 pipelines. For the right temporal cortex ROI (right panel), colors range from blue, indicating a single pipeline, to red, indicating 844 pipelines.

The foregoing analysis investigated pipeline variability in the localization of left- and right hemisphere activation peaks. However, investigators may be more interested in the localization of peak activation within specific brain regions rather than an entire cerebral hemisphere. To explore pipeline variability within circumscribed ROIs, peak activation coordinates were extracted for each pipeline within ROIs comprising the right inferior frontal gyrus and the right temporal cortex. This analysis identified 223 unique activation peaks in the right inferior frontal gyrus and 197 unique peaks in the right temporal cortex. As displayed in Figure 5, activation peaks were distributed widely across the right inferior frontal gyrus. Peaks in the right temporal cortex were relatively concentrated toward the center of the region, but nevertheless extended to span nearly the entire anterior-posterior and inferior-superior axes of the mask.

In sum, the localization of activation peaks also revealed both consistency and variability across analysis pipelines. While many pipelines yielded peak hemispheric activation locations in a network of regions thought to be related to response inhibition (Aron et al., 2004), peak locations were scattered widely throughout these regions, as well as additional regions throughout much of the brain. Analysis of peak activation distribution within inferior frontal and temporal regions also revealed considerable variability in localization across pipelines.

### Analytic variability of activation significance

The previous analyses revealed substantial quantitative variation in analysis outcomes (i.e., activation strength and location) across pipelines. Analysis of statistically thresholded images revealed that qualitative analysis outcomes (i.e., activation significance) varied with respect to methodological decisions as well. The 6,912 statistical maps were thresholded and corrected for multiple comparisons using five strategies: three variants of a Monte Carlo procedure, as well as FDR and Gaussian RFT corrections (Table 3). These parameters yielded 34,560 unique thresholded maps for each contrast.

For the successful stop versus go contrast, the proportion of significantly activated voxels (excluding non-brain voxels) varied from 0 to 26.3%, with a median of 4.6%. Monte Carlo simulation with a single-voxel threshold of *p* < 0.01 proved to be the most liberal procedure, with a median of 12.8% of brain voxels activated. Monte Carlo simulation with single-voxel thresholds of *p* < 0.001 and *p* < 0.0001 yielded median activation proportions of 5.4 and 1.9%, respectively. Using FDR correction yielded a median activation proportion of 10.8%. RFT correction was the most conservative approach, with a median of 0.16% of brain voxels activated. Critically, all five thresholding methods aimed to control the whole-brain false positive rate at 5%. Thus, these results suggest that some thresholding approaches are far more conservative than others, even when targeting the same corrected false positive rate – a point that has been raised in previous studies (e.g., Lieberman and Cunningham, 2009) but that merits being repeated here.

**Figure 6:**
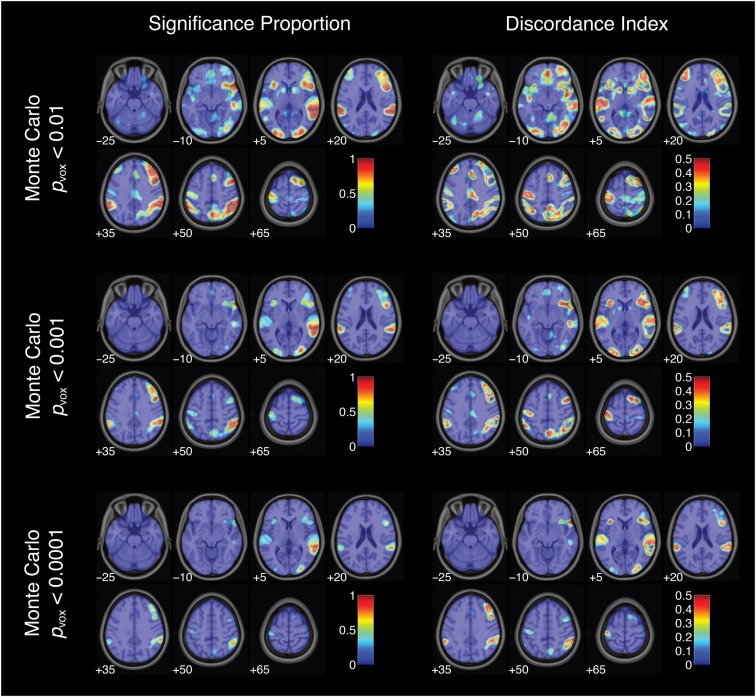
**Activation significance across analysis pipelines using three variants of a Monte Carlo thresholding procedure**. Significance proportion denotes the fraction of thresholded maps yielding significant activation for each voxel. Discordance index denotes the level of disagreement across threshold maps. Images are presented in neurological orientation, with the left hemisphere displayed on the left. Note that color scales range from 0 to 1 for significance proportion and from 0 to 0.5 for discordance index.

To characterize the likelihood of significant activation across all 34,560 thresholded maps, the proportion of pipelines yielding significant activation was computed for each voxel (Figure 6). This index did not reach 1 (or 100%) for any voxel for the successful stop versus go contrast. In other words, no voxels showed significant activation under all analysis and thresholding pipelines. However, some voxels consistently showed significant activation over nearly every analytic approach. The peak significance proportion in the right superior temporal gyrus reached 0.93. A subset of voxels in the right inferior frontal gyrus and right middle occipital gyrus also showed significant activation across a majority of pipelines, with peak significance proportions of 0.77 and 0.83, respectively. In contrast, many voxels deep within the arcuate fasciculus yielded significance proportions of zero: these voxels did not show significant activation under any combination of analytic and thresholding strategies. Somewhat paradoxically, voxels showing relatively consistent activation (i.e., high significance proportion indices) also exhibited relatively strong quantitative variability across analysis pipelines (i.e., high analytic range values; *R*^2^ = 0.64); analytic range values in the voxels of peak significance proportion in the right superior temporal gyrus, the right inferior frontal gyrus, and the right middle occipital gyrus were 8.13, 6.57, and 7.05 *Z*-units, respectively. Finally, nearly all voxels yielded non-zero significance proportions: 90.3% of brain voxels showed significant activation for at least some thresholded maps.

Thus, some voxels were significantly activated for nearly all analysis pipelines; others did not yield significant activation under any pipelines. However, some voxels yielded less consistent results across pipelines. This disagreement about qualitative analysis outcomes was assessed at each voxel using the discordance index:

discordance = minimum(significance proportion, 1 – significance proportion).

**Figure 7:**
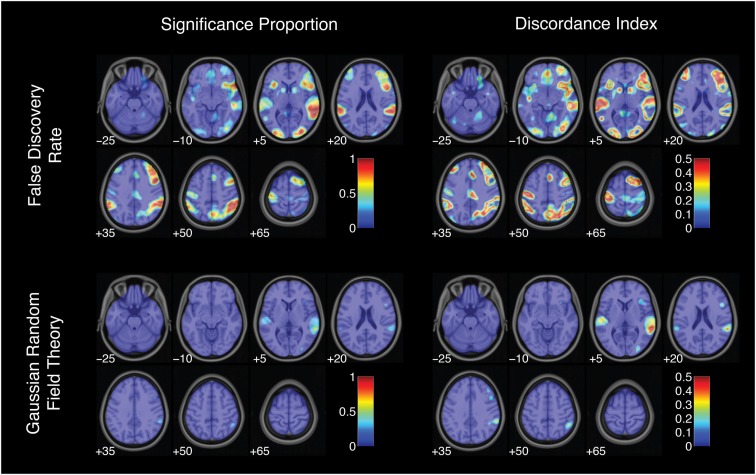
**Activation significance across analysis pipelines using false discovery rate and Gaussian random field theory error corrections**. Significance proportion denotes the fraction of thresholded maps yielding significant activation for each voxel. Discordance index denotes the level of disagreement across threshold maps. Images are presented in neurological orientation, with the left hemisphere displayed on the left. Note that color scales range from 0 to 1 for significance proportion and from 0 to 0.5 for discordance index.

This index ranged from 0 (when either 0 or 100% of analysis pipelines yielded significant activation) to 0.5 (when exactly 50% of pipelines yielded significant activation). Discordance indices were high, often reaching the theoretical maximum value of 0.5, in voxels surrounding regions of peak significance proportions (Figures 6 and 7). For example, voxels bordering the bilateral superior temporal gyrus and the right inferior frontal gyrus showed consistently high disagreement across analysis pipelines. These discordance rings around activation foci likely reflect the effects of differing spatial smoothing kernels on activation extent. Additional regions of disagreement included the precuneus (discordance index = 0.50), anterior cingulate cortex (discordance index = 0.44), and middle cingulate gyrus (discordance index = 0.30).

Altogether, estimates of the spatial extent of significant activation and the proportion of thresholded maps showing significant activation revealed substantial flexibility across methodological strategies. Furthermore, regions showing strong disagreement across pipelines were observed throughout the brain, both in the neighborhood of peak significance proportions and in additional isolated clusters.

## Discussion

According to a mathematical model of bias in scientific research (Ioannidis, 2005b), the prevalence of false positive results in published reports increases with the flexibility of research outcomes. Research outcomes are flexible to the extent that (a) researchers have access to a broad range of experimental design and data analytic strategies and (b) different research strategies yield different research outcomes. A recent survey of methods used in the fMRI literature shows that research strategies are highly flexible across published studies, with nearly as many unique methodological pipelines as studies in the sample (Carp, 2012). However, the extent to which flexible research strategies translate into flexible research outcomes remains unclear. Thus, the present study sought to estimate the flexibility of research outcomes across a wide range of complete analysis pipelines applied to a single fMRI experiment.

The present results revealed both consistency and variability across analysis pipelines. Some results were highly stable across pipelines. For example, voxels in the right superior temporal gyrus, the right inferior frontal gyrus, and the right middle occipital gyrus showed significant activation for the successful stop versus go contrast for at least 77% of the 34,560 thresholded maps considered here. Thus, although quantitative responses (i.e., activation strength and location) in these regions proved variable across pipelines, their qualitative responses (i.e., activation significance) were quite stable. In other words, although we can be very confident that the level of stop-related activation in right inferior frontal gyrus is greater than zero, there is much greater uncertainty about the strength of this activation or its precise location within the inferior frontal gyrus. These observations are consistent with the view that the right inferior frontal gyrus is specialized for inhibitory control (e.g., Aron et al., 2004). These results also largely uphold the conclusions of the original stop-signal experiment by Aron and colleagues (2007).

However, results also varied considerably from one pipeline to another. Estimates of activation strength were highly variable across analytic pipelines: in regions of peak overall activation, significance estimates varied by over 8 *Z*-units. The localization of peak activation also proved to be strongly pipeline-dependent. Hundreds of unique peak coordinates were observed for each contrast, with peak locations scattered throughout much of the brain. For example, the contrast of failed stop trials versus baseline yielded activation peaks in 83 of the 128 regions defined by the AAL atlas. Finally, estimates of statistical significance showed substantial variability across pipelines as well. For example, for the successful stop versus go contrast, the proportion of activated brain voxels ranged across pipelines from 0 to 26.3%. While some voxels were consistently activated, others showed strong disagreement across analysis pipelines.

The flexibility of research outcomes illustrated here, along with mathematical models linking flexible research methods with elevated false positive rates (Ioannidis, 2005b), suggests that the risk of false positive results in fMRI research may be greater than expected. Nearly every voxel in the brain showed significant activation under at least one analysis pipeline. In other words, a sufficiently persistent researcher determined to find significant activation in virtually any brain region is quite likely to succeed. By the same token, no voxels were significantly activated across all pipelines. Thus, a researcher who hopes not to find any activation in a particular region (e.g., to rebut a competing hypothesis) can surely find a methodological strategy that will yield the desired null result. If investigators apply several analysis pipelines to an experiment and only report the analyses that support their hypotheses, then the prevalence of false positive results in the literature may far exceed the nominal rate.

It is important to note, however, that analytic flexibility only translates into elevated false positive rates when combined with selective analysis reporting. In other words, if fMRI researchers reported the results of all analysis pipelines used in their studies, then the flexibility documented here would not be problematic. To the author’s knowledge, there is no evidence that fMRI researchers actually engage in selective analysis reporting. But researchers in other fields do appear to pursue this strategy. Surveys comparing research protocols to published articles show that a majority of randomized clinical trials add, omit, or replace study outcome variables – and, critically, that investigators are more likely to report significant outcomes than non-significant outcomes (Chan et al., 2004a,b; Dwan et al., 2008; Mathieu et al., 2009). Similarly, studies of putative brain volume abnormalities in patients with mental health disorders report far more positive results than would be expected given their power to detect such effects, likely reflecting the selective reporting of favorable analysis outcomes (Ioannidis, 2011). Thus, if fMRI researchers behave like researchers in other fields, then the methodological flexibility illustrated here would indeed imply an elevated rate of false positive results in the fMRI literature.

Critically, selective analysis reporting may occur without the intention or even the awareness of the investigator. For example, if the results of a new experiment do not concord with prior studies, researchers may adjust analysis parameters until the “correct” results are observed. Researchers may also elect not to describe the results of all analysis pipelines due to space limitations in journal articles or to preserve the narrative flow of a manuscript. Finally, researchers may simply not be aware of the risks posed by selective analysis reporting. Thus, although the practice of selective analysis reporting is deeply problematic, it need not reflect any malice on the part of the researchers who engage in it.

It is also important to note that bias related to analytic flexibility and selective analysis reporting is not unique to fMRI research. Indeed, previous studies have argued that selective analysis reporting can lead to false positive results in studies of randomized controlled trials (Chan et al., 2004a,b), brain volume abnormalities in psychiatric disorders (Ioannidis, 2011), and in the broader research literature (Ioannidis, 2005b). Selective analysis reporting can contaminate research results in any empirical field that allows for multiple analytic approaches – in other words, for nearly all empirical studies.

### Limitations

Although the present study revealed a wide range of research outcomes for a single experiment, the approach used here likely underestimated the true flexibility of fMRI analysis methods. The present study considered two to four parameters for each analysis step, but many more parameters appear in the literature. For example, while this study considered three normalization targets, a methodological survey of recent fMRI studies (Carp, 2012) revealed a range of at least ten unique normalization targets. Similarly, while high-pass filtering cutoffs ranged from 0.33 to 750 s in this methodological survey, the present study only considered two filtering parameters: a cutoff of 128 s or no temporal filtering.

In addition, a number of key analysis steps were not considered in the present study. For example, the present approach did not investigate the effects of different strategies for coregistration between structural and functional images, for brain extraction and segmentation, for signal normalization, or for physiological noise reduction – e.g., as implemented in RETROICOR (Glover et al., 2000) or PHYCAA (Churchill et al., 2012c). Similarly, this study did not consider tools for the correction or deletion of noisy slices, brain volumes, or subjects, which may exert strong effects on analysis outcomes (Tohka et al., 2008; Power et al., 2012).

Furthermore, this study relied largely on analysis steps implemented in the SPM 8 software library. However, fMRI researchers use several versions of SPM and a wide variety of different software packages, with 32 unique libraries reported across a recent survey of fMRI studies (Carp, 2012). Studies may also combine analysis routines from multiple libraries, further increasing the flexibility of methodological approaches in the fMRI literature. This flexibility across software options may also contribute to analytic flexibility. Different libraries may offer different strategies for the same analysis step. Further, even if multiple packages attempt to implement the same algorithms, ambiguities inherent in the translation from natural and mathematical language to computer programs may nonetheless result in differences between implementations (Ince et al., 2012). Indeed, informal comparisons suggest that choices of software package can have substantial effects on analysis outcomes (Poline et al., 2006).

The present study also relied on a relatively small sample size of 13 subjects. This sample size may have rendered many of the pipelines underpowered to detect true effects, leading to high rates of false negative results. However, the median sample size of single-group fMRI studies is approximately 15 subjects (Carp, 2012). Thus, while the present study is likely to be underpowered, it is also about as underpowered as the typical study of its kind. Thus, analytic flexibility in this sample is likely to be broadly representative of typical fMRI studies. Nevertheless, future studies should repeat this analysis using larger sample sizes to determine how or whether estimates of methods variability change with statistical power.

In addition, the extent to which the analysis pipelines investigated in this experiment resemble the true distribution of pipelines in the research literature is unclear. To the extent that the distribution of pipelines considered here differs from the distribution in the research literature, the present study may either underestimate or overestimate the true flexibility of analysis outcomes. For example, one third of the pipelines considered here estimated parameters for spatial normalization using the unified segmentation approach of SPM 8. But perhaps fewer or more than one third of published fMRI reports appear to use this approach. Analogously, all of the pipelines considered here included some form of correction for multiple comparisons. But a substantial fraction of published studies appear not to use such corrections (Carp, 2012). Thus, the pipelines examined in this study may not be fully representative of the pipelines used in published reports. However, because many published studies do not explicitly report which analysis steps and parameters were used (Carp, 2012), it is challenging to determine the true distribution of analysis pipelines in the literature. Future studies should continue to investigate the prevalence of different analysis pipelines and the effects of these pipelines on research outcomes.

Finally, it is important to note that the present study did not address the issue of which analysis pipelines should be used. Instead, this study merely sought to estimate the flexibility of research results across pipelines. As described in the Introduction, many previous studies have considered the problem of pipeline optimization (e.g., Strother et al., 2004; Churchill et al., 2012a,b).

### Recommendations

What steps can investigators take to mitigate the risk of false positive results posed by flexible analysis methods in fMRI studies? As discussed above, the true range of fMRI methods cannot be estimated unless research reports describe analysis pipelines in detail. Thus, researchers should thoroughly describe the analysis methods chosen, as well as the reasoning behind those choices. Unfortunately, many published reports do not explicitly describe critical design and analysis decisions (Carp, 2012). Standardized reporting guidelines may help fMRI researchers to communicate methodological choices in greater detail. Such guidelines, which have been widely adopted by academic journals that publish studies of randomized controlled trials (Moher et al., 2001), diagnostic accuracy (Bossuyt et al., 2003), and observational epidemiology (von Elm et al., 2007), can significantly improve the quality of methods reporting (Plint et al., 2006). Although no consensus guidelines for the reporting of fMRI methods exist at present, the reporting recommendations by Poldrack et al. (2008) provide a useful starting point.

Flexibility in research methods may be particularly problematic when it is undisclosed (Simmons et al., 2011). For example, a hypothetical group of investigators might analyze an experiment using a range of methodological strategies and discover that only a few strategies yield positive results. If these investigators only report the pipelines that favor their hypotheses, then readers may not realize that the results of the experiment depend on (perhaps arbitrary) methodological decisions. Thus, it is critical that fMRI researchers report all analysis pipelines used in the course of data analysis, whether or not those pipelines yielded results favorable to the researchers’ hypotheses. For example, if a research team initially used a canonical hemodynamic response function to model activation time series but later opted to use a finite impulse response basis set instead, the results of both strategies should be described in full. Similarly, if researchers discover that a contrast of interest yields significant activation using Monte Carlo correction but not using FDR correction, both sets of activation maps should be reported. If investigators only describe a single analysis pipeline, they should also certify that no additional pipelines were used. Finally, reviewers can work to mitigate selective analysis reporting as well. Indeed, Simmons and colleagues (2011) argue that “reviewers should require authors to demonstrate that their results do not hinge on arbitrary analytic decisions.” If authors fail to indicate that they have fully described all analysis pipelines, reviewers should require them to do so; if reviewers suspect that critical results may depend on arbitrary methodological decisions, they may ask authors to defend their choices or to report the results of equally valid decisions.

Sharing data and analysis code may also help to unmask hidden flexibility in the analysis of fMRI experiments. If raw data for an experiment are freely available, then interested readers may reanalyze experiments on their own, searching out the analytic boundary conditions of reported results. Several promising data sharing initiatives focusing on resting-state imaging (the 1000 Functional Connectomes Project)^4^, structural imaging (the Open Access Series of Imaging Studies database)^5^, and task-based paradigms (the Open fMRI database)^6^ are currently underway. Data from the present study were drawn from the Open fMRI database; analysis code is freely available online (see text footnote 3).

False positive results driven by analytic flexibility may also be mitigated by curtailing the range of available methodological strategies. For example, investigators may develop standardized analysis pipelines that they apply to all of their experiments. Researchers may also simply adhere to the default options in their software packages of choice. However, while both of these approaches have the potential to reduce analytic flexibility and selective analysis reporting, they may not yield optimal analysis pipelines. Continued methodological research can also shrink the space of analytic approaches. For example, Sladky et al. (2011) argue that studies should perform slice-timing correction (but see also Poldrack et al., 2011, pp. 41–42); Purdon and Weisskoff (1998) suggest that studies should correct for temporal autocorrelation; and Lund et al. (2005) argue that studies should include head motion regression. Following these recommendations alone would reduce the number of pipelines in the present study from 6,912 to 1,296; additional research on optimal procedures and parameters may further reduce experimenter degrees of freedom. Pipeline optimization tools developed by Strother and colleagues can also be used to reduce analysis flexibility (e.g., Strother et al., 2004; Churchill et al., 2012a,b). These tools automatically identify the analysis pipelines that maximize reproducibility and prediction metrics estimated from the data on a subject-by-subject basis. Thus, using these methods reduces the risk that investigators might use a range of analysis pipelines and selectively report those that yield favorable results.

While these recommendations have the potential to reduce bias due to analytic flexibility and selective analysis reporting, they do not address other sources of error and bias. For example, while reporting the results of all analysis pipelines would (by definition) eliminate selective analysis reporting, it does not guarantee that any of the reported pipelines is optimal. As noted above, continued research on pipeline optimization may help to resolve this problem. In addition, none of these recommendations can address the problems of intentional misrepresentation or fraud. The voluntary guidelines described here cannot prevent researchers from covertly engaging in selective analysis reporting and claiming not to have done so – or from manipulating or fabricating results. Fortunately, though, relatively few scientists appear to engage in outright fraud (John et al., 2012).

## Conclusion

The present study reveals both consistency and flexibility in the analysis of fMRI experiments. While some research outcomes were relatively stable across analysis pipelines, others varied widely from one pipeline to another. Given the extent of this variability, a motivated researcher determined to find significant activation in practically any brain region will very likely succeed – as will another researcher determined to find null results in the same region. To mitigate the effects of this flexibility on the prevalence of false positive results, investigators should either determine analysis pipelines *a priori* or identify optimal pipelines using data-driven metrics. If researchers use multiple pipelines to analyze a single experiment, the results of all pipelines should be reported – including those that yielded unfavorable results. If implemented, these steps could significantly improve the reproducibility of research in the fMRI literature.

## Conflict of Interest Statement

The author declares that the research was conducted in the absence of any commercial or financial relationships that could be construed as a potential conflict of interest.

## Footnotes

^1^http://www.openfmri.org

^2^http://www.restfmri.net

^3^https://github.com/jmcarp/fmri-pipe

^4^http://fcon_1000.projects.nitrc.org

^5^http://www.oasis-brains.org

^6^http://openfmri.org
